# Enabling next‐generation therapies: A foreword to a special issue on nanotechnology in medicine

**DOI:** 10.1002/btm2.10678

**Published:** 2024-05-23

**Authors:** Josué Sznitman

**Affiliations:** ^1^ Department of Biomedical Engineering Technion – Israel Institute of Technology Haifa Israel

The Spring of 2022 coincided with a long‐awaited return of the conference series on Nanotechnology in Medicine (Calabria, Italy), chaired on this occasion by Dr Milica Radisic (University of Toronto) and Dr Victor Shahin (University of Münster) under the auspices of Engineering Conference International (ECI). To celebrate the main highlights of such event, the special issue of *Bioengineering & Translational Medicine* (Volume X, Issue X) brings together a curated collection of stimulating contributions from plenary, keynote, and invited speakers of the conference under the unifying theme of “enabling next‐generation therapies.”

The third edition of this conference provided an intimate yet lively scientific forum whose purpose expanded upon the scope of the past two previous editions of the conference series (see, e.g., *Bioengineering & Translational Medicine* Vol. 4, Issues 2 & 3, 2019) in discussing recent research developments in the aforementioned field. Among the leading topics emphasized in this 2022 edition of the conference were (i) a deepening of the mechanistic understanding of biodistribution of systematically targeted nanoparticles (NPs), (ii) exploring the effects of mechanical environments of tissues and cells, (iii) the use of tissue and *organ‐on‐chip* (OoC) models in the studies of NP distribution and toxicity, (iv) generating an improved mechanistic understanding of the factors necessary to control in vivo NP targeting; and (v) exploiting such understanding to generate highly effective nanotechnologies for the early detection, imaging, and treatment of human diseases.

In this short editorial, we briefly take the opportunity to highlight a few contributions of interest that mark the special issue. Resonating with the timeliness of the COVID‐19 pandemic, Lu et al. (https://doi.org/10.1002/btm2.10581) discuss recent advances in *heart‐on‐a‐chip* platforms for elucidating SARS‐CoV‐2 pathogenesis, including the potential mechanisms that drive heart failure whereby viral infection induces myocardial dysfunction, with an outlook toward more advanced models for disease modeling and pharmacological discovery. Continuing in the area of OoC, Spitz et al. (https://doi.org/10.1002/btm2.10604) provide an overview of recent OoC advances in the field of neurodegenerative diseases (NDDs) directed toward non‐invasive sensing strategies encompassing electrical, electrochemical and optical sensors. Motivated by the lack of insufficient predictive validity of animal‐based disease models for clinical trials, the authors discuss promising on‐ and integrable off‐chip sensing OoC strategies applicable to NDD research to advance the translational value of microphysiological systems in preclinical settings.

In parallel, Ramezani et al. (https://doi.org/10.1002/btm2.10652) discuss the potential of dye supramolecular assemblies for broad applications such as photoacoustic and fluorescence imaging, as well as photothermal and photodynamic therapies. There, the authors expand on emerging applications of dyes as drug‐stabilizing agents used together with aggregator molecules to form stable NPs in view of further translational in vivo endpoints for clinical use. In the area of joint diseases (e.g., osteoarthritis), intra‐articular delivery of drugs to cartilage remains an unresolved challenge due to their rapid clearance within joints. Here, Gonzales et al. (https://doi.org/10.1002/btm2.10612) discuss the development of new cationic nanocarriers with variable charge that form reversible electrostatic interactions with the anionic extra‐cellular matrix of cartilage. The authors present results both in vitro and in mouse cartilage explants supporting a proof‐of‐concept study with the transport of cationic, branched poly‐l‐lysine nanocarriers through negatively charged cartilaginous tissues that can promote deeper penetration and prolongment of drug retention. As a final example, in the areas of systemic delivery in the cardiovascular system, the review of Asaad et al. (https://doi.org/10.1002/btm2.10669) discusses interactions of several key NP types (e.g., polymeric, ceramic, silica, dendrimers, and metallic) on circulating platelets in blood, with a focus on the physicochemical parameters that may modulate the therapeutic potential of such NPs when designing safe and effective therapies that can be translated into clinical practice.

### PEER REVIEW

The peer review history for this article is available at https://www.webofscience.com/api/gateway/wos/peer-review/10.1002/btm2.10678.

